# Effects of supplemental citrulline on thermal and intestinal morphology parameters during heat stress and feed restriction in growing pigs

**DOI:** 10.1093/jas/skae120

**Published:** 2024-05-30

**Authors:** Sara K Kvidera, Edith J Mayorga, Carrie S McCarthy, Erin A Horst, Megan A Abeyta, Lance H Baumgard

**Affiliations:** Department of Animal Science, Iowa State University, Ames, Iowa 50011, USA; Department of Animal Science, Iowa State University, Ames, Iowa 50011, USA; Department of Animal Science, Iowa State University, Ames, Iowa 50011, USA; Department of Animal Science, Iowa State University, Ames, Iowa 50011, USA; Department of Animal Science, Iowa State University, Ames, Iowa 50011, USA; Department of Animal Science, Iowa State University, Ames, Iowa 50011, USA

**Keywords:** intestinal health, mucosal surface area, pair-feeding

## Abstract

Study objectives were to characterize the effects of citrulline (CIT) on physiological and intestinal morphology metrics during heat stress (HS) and feed restriction. Forty crossbred gilts (30 ± 2 kg body weight [BW]) were assigned to one of five treatments: (1) thermoneutral (TN) fed ad libitum (AL) with control (CON) supplement (TNAL; *n* = 8), (2) TN pair-fed (PF) with CON (PF-CON; *n* = 8), (3) TN PF with CIT (PF-CIT; *n* = 8), (4) HS AL with CON (HS-CON; *n* = 8), and (5) HS AL with CIT (HS-CIT; *n* = 8). During the period (P) 1 (7 d), pigs were in TN conditions (23.6 °C) and fed AL their respective supplemental treatments. During P2 (2.5 d), HS-CON and HS-CIT pigs were fed AL and exposed to cyclical HS (33.6 to 38.3 °C), while TNAL, PF-CON, and PF-CIT remained in TN and were fed either AL or PF to their HS counterparts. Citrulline (0.13 g/kg BW) was orally administered twice daily during P1 and P2. HS increased rectal temperature (Tr), skin temperature (Ts), and respiration rate (RR) relative to TN pigs (0.8 °C, 4.7 °C, and 47 breaths/min, respectively; *P* < 0.01). However, HS-CIT had decreased RR (7 breaths/min, *P* = 0.04) and a tendency for decreased Tr (0.1 °C, *P* = 0.07) relative to HS-CON pigs. During P2, HS pigs had decreased feed intake (22%; *P* < 0.01) and a tendency for decreased average daily gain (*P* = 0.08) relative to TNAL pigs, and by experimental design, PF pigs followed this same pattern. Circulating lipopolysaccharide-binding protein tended to be decreased (29%; *P* = 0.08) in PF relative to TNAL pigs and was increased (41%; *P* = 0.03) in HS compared to PF pigs. Jejunum villus height was decreased in PF relative to TNAL pigs (15%; *P* = 0.03); however, CIT supplementation improved this metric during feed restriction (16%; *P* = 0.10). Jejunum mucosal surface area decreased in PF (16%; *P* = 0.02) and tended to decrease in HS (11%; *P* = 0.10) compared to TNAL pigs. Ileum villus height and mucosal surface area decreased in HS compared to TNAL pigs (10 and 14%, respectively; *P* ≤ 0.04), but both parameters were rescued by CIT supplementation (*P* ≤ 0.08). Intestinal myeloperoxidase and goblet cell area remained similar among treatments and intestinal segments (*P* > 0.24). In summary, CIT supplementation slightly improved RR and Tr during HS. Feed restriction and HS differentially affected jejunum and ileum morphology and while CIT ameliorated some of these effects, the benefit appeared dependent on intestinal section and stressor type.

## Teaser

Citrulline supplementation appears beneficial in alleviating the physiological strain induced by a thermal load and improving some parameters of intestinal morphology during heat stress and feed restriction.

## Introduction

Heat stress (**HS**) is a severe economic burden to animal agriculture; costing approximately $1 billion annually in the swine industry alone (St. Pierre et al., 2003; Ross et al., 2015). Many of the negative consequences of HS on animal health and productivity appear to be instigated by gastrointestinal tract (**GIT**) hyperpermeability (Baumgard and Rhoads, 2013; Mayorga et al., 2020). The prevailing thought is that HS causes peripheral (skin) vasodilation to maximize radiant heat dissipation, and this is accompanied by coordinated visceral vasoconstriction to prevent hypotension (Kregel et al., 1988; Hall et al., 2001; Burhans et al., 2022). This leads to GIT hypoxia, to which enterocytes are particularly sensitive, and consequently, intestinal barrier function is compromised (Hall et al., 1999; Lambert et al., 2002). Disrupted GIT homeostasis during HS can lead to luminal contents (e.g., endotoxins and other antigens) infiltrating into local, portal, and systemic circulation, which causes immune stimulation and subsequent inflammation (Graber et al., 1971; Leon, 2007). Furthermore, feed restriction (heat-stressed animals voluntarily become anorexic; Baumgard and Rhoads, 2013) alone can negatively impact gut barrier function in pigs (Pearce et al., 2013a) and various other species (Rodriguez et al., 1996; Welsh et al., 1998; Boza et al., 1999; Kvidera et al., 2017a). Thus, nutritional strategies aimed to assist the GIT epithelial barrier during stress are of practical interest.

Citrulline (**CIT**) is a non-essential amino acid produced by enterocytes of the small intestine and is a precursor for Arg synthesis (Bahri et al., 2013). Supplemental CIT improves Arg status, sometimes better than Arg itself (Agarwal et al., 2017), largely because dietary Arg is metabolized by the intestine and liver (Cynober, 1994). Because the enterocytes of the small intestine are the main site of CIT production, both CIT and Arg easily become limited during situations that compromise gut function (e.g., intestinal resection, HS). Citrulline and Arg are also important precursors for nitric oxide (**NO**) synthesis and regulation, which becomes critical during immune activation and conditions requiring vasodilation (i.e., HS). Arg has been labeled a conditionally essential amino acid during stresses such as sepsis or intestinal resection (Osowska et al., 2004, 2008; Wijnands et al., 2015). This is likely due to its role in NO synthesis, immune regulation, wound healing, and insulin sensitivity (Pekarova and Lojek, 2015; Wijnands et al., 2015; Varasteh et al., 2018). Furthermore, supplemental CIT improves markers of intestinal integrity in various models of GIT injury (Chapman et al., 2012; van Wijck et al., 2014; Antunes et al., 2016), potentially due to its ability to restore NO function and visceral microcirculation (Wijnands et al., 2012). During HS, supplementing CIT has been shown to decrease body temperature and influence the antioxidant status and the heat shock response in chickens and laying hens (Chowdhury et al., 2017, 2021b; Uyanga et al., 2020, 2021). Furthermore, CIT supplementation reduced the respiration rate (RR) in lactating sows and decreased the preweaning mortality rate in piglets during the summer (Liu et al., 2019). Thus, CIT shows promise as a tool to help reduce the physiological strain stemming from HS; however, little is known about how (or if) CIT influences the intestinal mucosal barrier during HS in production animals. Therefore, objectives were to evaluate the effects of CIT supplementation on physiological and gut health parameters during HS and nutrient restriction in growing pigs.

## Materials and Methods

### Animals

All procedures were reviewed and approved by the Iowa State University Institutional Animal Care and Use Committee (approved IACUC #11-15-8128-S). Forty crossbred female pigs [30 ± 2 kg body weight (**BW**)] were blocked by initial BW and assigned to one of five environmental-supplemental treatments: (1) thermoneutral (**TN**) ad-libitum (**AL**) control (**TNAL**; *n* = 8), (2) TN pair-fed (**PF**) control (**PF-CON**; *n* = 8), (3) TN PF with CIT supplement (**PF-CIT**; *n* = 8), (4) HS AL control (**HS-CON**; *n* = 8), and (5) HS AL with CIT supplement (**HS-CIT**; *n* = 8). Pigs were housed in individual pens (51 × 221 cm) in one of two rooms in the Discovery Wing at the Iowa State University Swine Nutrition Farm. Each crate was equipped with a stainless-steel feeder and a nipple drinker. Pigs were fed a diet formulated to meet or exceed the predicted requirements for growing pigs (NRC, 2012; Table 1) for essential amino acids, minerals, and vitamins. Water was provided AL during the entire experiment.

**Table 1.** Ingredients and composition of diet (as-fed basis)

Ingredient	%
Corn	62.70
Soybean meal, CP 46%	24.16
Corn DDGS^1^	10.25
Monocalcium phosphate	0.38
Lysine HCl	0.37
dl-Methionine	0.08
Threonine	0.06
Limestone	1.32
NaCl	0.51
Vitamin-mineral premix^2^	0.15
Phytase^3^	0.03

^1^Corn distillers dried grains with solubles.

^2^Vitamin mineral premix provided the following (per kilogram diet): 9,921 IU/kg of vitamin A, 1,819 IU/kg of vitamin D3, 53 IU of vitamin E, 0.03 mg of vitamin B12, 2.6 mg of menadione, 5.0 mg of riboflavin, 20 mg of D-Pantothenic acid, 25 mg of niacin, 2.2 mg of ethoxyquin, 132 mg of Fe (ferrous sulfate), 132 mg of Zn (zinc sulfate), 60 mg of Mn (manganous oxide), 24 mg of Cu (copper chloride), 0.90 mg of I (calcium iodate); and 0.20 mg of Se (sodium selenite).

^3^Ronozyme (500 FTU/kg), DSM Nutritional Products Ltd., Basel, Switzerland.

### Experimental design

Pigs were allowed to acclimate (3 to 4 d) to individual crates and subjected to TN conditions (23.6 ± 0.1 °C, 33.6% ± 0.1% relative humidity) with a 15:9 h light–dark cycle. Following acclimation, the study was divided into two experimental periods. During period 1 (**P1**; 7 d), pigs were fed ad-libitum and kept in TN conditions for the collection of baseline body temperature indices and production parameters. During period 2 (**P2**; 2.5 d), pigs in HS-CON and HS-CIT treatments were allowed to eat ad-libitum and were exposed to cyclical HS with temperatures averaging 33.6 °C at night (from 1901 to 0659 hours) and 38.3 °C during the day (from 0700 to 1900 hours): (day 1: 37.8 ± 0.1 °C, 25.4% ± 0.1% relative humidity during the day and 33.6 ± 0.1 °C, 26.5% ± 0.1% relative humidity at night; day 2: 38.7 ± 0.1 °C, 24.4% ± 0.1% relative humidity during the day and 33.5 ± 0.1 °C, 27.6% ± 0.1% relative humidity at night; day 3: 38.6 ± 0.1 °C, 27.4% ± 0.1% relative humidity during the day). Both room temperature and humidity were monitored and recorded every 5 min by four data loggers (Lascar EL-USB-2-LCD, Erie, PA) located in different quadrants of each room.

During P2, pigs assigned to TNAL treatment remained in TN conditions (23.8 ± 0.1 °C, 41.1% ± 0.1% relative humidity) and were fed ad-libitum. Pigs in PF-CON and PF-CIT treatments also remained in TN conditions but were PF to their HS counterparts to evaluate the direct effects of HS while eliminating the confounding effects of dissimilar nutrient intake. In brief, feed intake (**FI**) in P1 was averaged for each pig and used as a baseline. During days 1 and 2 of P2, the decrease in FI in HS pigs was calculated daily as a percentage of FI reduction relative to P1. The percentage of FI reduction was averaged for all pigs in the HS treatments per day of heat exposure and applied individually to the baseline of each pig in the PF treatments. The daily amount of feed provided was divided into three equal portions during P2 (0600, 1200, and 1800 hours) to minimize physiological changes associated with gorging and fasting. On day 3 of P2, pigs were sacrificed at 1800 hours; hence, this day’s FI calculations comprised 12 h. Therefore, PF animals were fed an average of their heat-stressed counterparts on day 3 of P2 12 h intake. Throughout the experiment, pigs assigned to the PF treatments lagged 1 d behind TNAL and HS pigs to allow for pair-feeding calculations. This pair-feeding technique has been extensively utilized previously (Pearce et al., 2013a; Sanz Fernandez et al., 2015a, b).

Supplements were fed twice daily (0600 and 1800 hours) during P1 and P2. Pigs received 20 g of cookie dough (DoBiz Foods, LLC, Ames, IA) either without supplement (**CON**; TNAL, PF-CON, HS-CON) or with 0.13 g/kg BW L-CIT (99.3% purity, MP Biomedicals, LLC, Cat. No. 101397, Lot No. QR10956, Santa Ana, CA) homogenized within the cookie dough (PF-CIT and HS-CIT). The CIT daily dose (0.26 g/kg BW) was selected based on studies by Osowska et al. (2004), Ananthakrishnan et al. (2009), and Fike et al. (2015). Supplements were not provided at 1800 hours on day 3 of P2, which was immediately prior to euthanasia. All supplements were stored at 4 °C until administration.

### Data collection and sampling

FI was measured daily at 0600 hours during P1 and P2 as feed disappearance. BW was obtained at 0900 hours 2 d prior to acclimation, and this weight was used to calculate the dose of CIT for each pig allocated to a CIT treatment. BWs were obtained at 1800 hours on the last day of acclimation, day 7 of P1, and immediately before euthanasia. Average daily gain (**ADG**) and feed efficiency (gain to feed ratio; **G:F**) were calculated for P1 and P2.

During P1, body temperature indices (rectal temperature [**Tr**], skin temperature [**Ts**], and RR) were obtained once daily at 1800 hours. During P2, Tr, Ts, and RR were obtained three times daily at 0600, 1200, and 1800 hours. Rectal temperatures were measured using a digital thermometer (Digital Thermometer with Flex Tip, Jorgensen Labs Inc., Item #J0134F). Skin temperature was measured on the rump using an infrared thermometer (IRT207: The Heat Seeker 8:1 Mid-Range Infrared Thermometer, General Tools, New York, NY). RR was determined by counting flank movements in a 15-s interval and multiplying by four to obtain breaths per minute.

Pigs were restrained using a top-jaw snare, and blood samples were obtained by jugular venipuncture into evacuated K2EDTA tubes for plasma collection (10 mL each; BD Vacutainer, Franklin Lakes, NJ) at 1800 hours on the final day of acclimation, day 7 of P1, and immediately before euthanasia. Plasma was harvested by centrifugation at 4 °C and 1,500 × *g* for 15 min, aliquoted, and stored at −20 °C until analysis.

Pigs were euthanized at 1800 hours on day 3 of P2 using the captive bolt technique followed by exsanguination. A 38 cm section of the jejunum was collected approximately 95 cm distal from the pyloric sphincter. A 38 cm segment of the ileum was collected approximately 18 cm proximal to the ileocecal junction. A 38 cm segment of the colon was collected approximately 30 cm proximal to the rectum. All segments were flushed with cold phosphate-buffered saline to remove intestinal contents. A transversal section measuring ~2.5 cm was taken from the middle portion of each ileum, jejunum, and colon segment, fixed in 10% neutral buffered formalin for 24 h, and then transferred into 70% ethanol.

### Laboratory analyses

Plasma lipopolysaccharide-binding protein (**LBP**) was determined using a commercially available kit validated in our laboratory (Hycult Biotech, Uden, the Netherlands). For histological analysis of intestinal tissue, fixed jejunum, ileum, and colon samples were submitted to the Iowa State University Veterinary Pathology Laboratory for sectioning and staining. A periodic acid-Schiff (**PAS**) stain was utilized to quantify the goblet cell (**GC**) area. A hematoxylin and eosin (**H&E**) stain was utilized to quantify morphological measurements. For the myeloperoxidase (**MPO**) immunohistochemistry stain, primary and secondary antibodies were Dako Polyclonal anti-MPO and Multilink/HRP, respectively. The stain for MPO measurement was Nova red, and slides were counterstained with 50% hematoxylin. For each stain type, one slide per pig per tissue was generated. Using a microscope (Leica DMI3000 B Inverted Microscope, Bannockburn, IL) with an attached camera (QImaging 12-bit QICAM Fast 1394, Surrey, BC), five images per intestinal section were obtained at 50 × (for H&E and PAS stains) and 200 × (for MPO stain) magnification. All image processing and quantification were completed using ImageJ 1.48v (National Institutes of Health, USA). The PAS stain was measured using the Image J color deconvolution tool with H PAS vector. GC area was expressed as a percentage of the total mucosal area stained by PAS. The MPO area was calculated with the color deconvolution tool using the H&E DAB vector and is expressed as the percent MPO stain of the epithelial area. Villus (**v.**) height was measured from the tip to the villus-crypt interface. Villus width was measured at mid-villus height. Crypt (**c.**) depth was measured from the villus-crypt opening to the lamina propria. Crypt width was measured at the villus-crypt interface level. A mucosal surface area estimate was obtained using the mucosal-to-serosal amplification ratio M as previously reported by Kisielinski et al. (2002), where


$$\begin{array}{l} M=\frac{\left(v.\;width\times v.\;length\right)+{\left(\frac{v.\;width}{2}+\frac{c.\;width}{2}\right)}^{2}-{\left(\frac{v.\;width}{2}\right)}^{2}}{{\left(\frac{v.\;width}{2}+\frac{c.\;width}{2}\right)}^{2}} \end{array}$$

### Statistical analysis

Data were analyzed using PROC MIXED in SAS 9.4 (SAS Institute Inc., Cary, NC). For production parameters (FI, ADG, and G:F) and circulating LBP, P1 and P2 were analyzed separately. The effects of block and treatment were included in the model. For production parameters, BW obtained at the end of acclimation was used as a covariate. For LBP, values obtained from the blood sample taken at 1800 hours on the final day of acclimation were used as a covariate. For body temperature indices, average P1 values for Tr, Ts, and RR were used as a covariate, and effects of block, treatment, time, and treatment by time interaction were assessed using repeated measures with an autoregressive covariance structure. For intestinal histology measurements, the effects of block and treatment were analyzed. For all data, preplanned contrasts of TNAL vs. PF (PF-CON and PF-CIT), TNAL vs. HS (HS-CON and HS-CIT), PF (PF-CON and PF-CIT) vs. HS (HS-CON and HS-CIT), PF-CON vs. PF-CIT, and HS-CON vs. HS-CIT were included. Data are presented as least squares means, and statistical significance was defined as *P* ≤ 0.05 and a tendency was defined as 0.05 < *P* ≤ 0.10.

## Results

**Figure 1.**
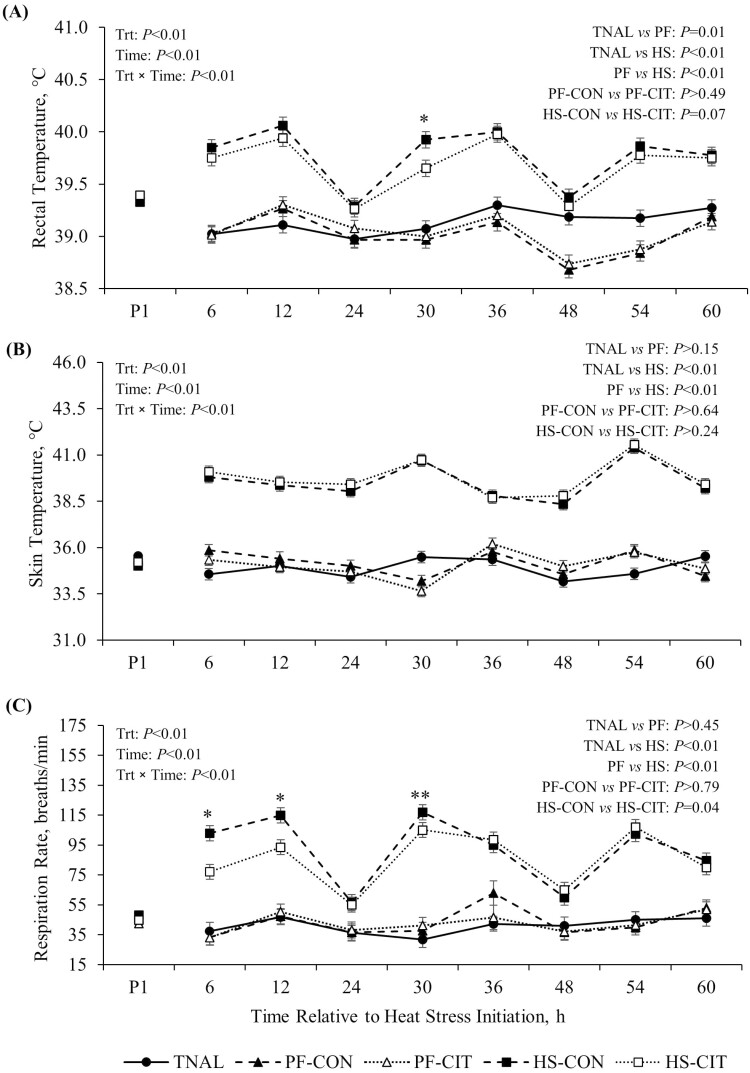
Effects of heat stress (**HS**) or pair-feeding (**PF**) and control (**CON**) or citrulline (**CIT**) supplement on (A) rectal temperature, (B) skin temperature, and (C) respiration rate. TNAL, thermoneutral ad-libitum; PF-CON, pair-fed control; PF-CIT, pair-fed citrulline; HS-CON, heat stress control; HS-CIT, heat stress citrulline. The HS protocol employed cyclical room temperatures ranging from 33.6 °C at night (from 1901 to 0659 hours) to 38.3 °C during the day (from 0700 to 1900 hours). Data are represented as least squares means ± standard error of the mean. *Denotes a significant difference (*P* ≤ 0.05) between HS-CON and HS-CIT treatments. **Denotes a tendency (0.05 < *P* ≤ 0.10) for a difference between HS-CON and HS-CIT treatments.

During P2, pigs exposed to HS had increased Tr (0.8 °C), Ts (4.7 °C), and RR (47 breaths/min) relative to TNAL pigs (*P *< 0.01; Figure 1A, B, and C). PF animals had decreased rectal temperature (0.12 °C; *P *= 0.01; Figure 1A) relative to TNAL pigs, but Ts and RR were similar between PF and TNAL controls (*P* ≥ 0.15; Figure 1B and C). Interestingly, HS-CIT pigs had decreased RR (7 breaths/min; *P *= 0.04; Figure 1C) and a tendency for decreased Tr (0.1 °C; *P *= 0.07; Figure 1A) compared to HS-CON pigs.

There were no major production differences during P1 when comparing CON vs. CIT-supplemented pigs (*P* > 0.16; Table 2). During P2, pigs subjected to HS had decreased FI (22%; *P *< 0.01) and a tendency for decreased ADG (19%; *P *= 0.08; Table 3) relative to TNAL pigs. However, G:F remained similar between HS and TNAL pigs (*P* > 0.52; Table 3). By experimental design, PF pigs had similar FI compared to HS pigs (*P* > 0.43; Table 3). Additionally, ADG and G:F decreased (59% and 46%, respectively; *P *≤ 0.01) in PF relative to TNAL pigs. Supplemental CIT did not affect production metrics during HS or PF.

**Table 2.** Effects of citrulline supplementation on production parameters and circulating lipopolysaccharide-binding protein during period 1

Parameter	Treatment		SEM	*P*-value		
	CON	CIT		Trt	Day	Trt × day^1^
Performance						
FI, kg	1.99	2.06	0.04	0.22	0.01	0.20
ADG, kg	0.86	0.91	0.02	0.16	—	—
G:F	0.44	0.45	0.01	0.59	—	—
Inflammation						
LBP, µg/mL	13.34	12.02	0.96	0.34	—	—

^1^Treatment by day interaction.

CON, control-supplemented pigs; CIT, citrulline-supplemented pigs;

^Trt^treatment; FI, daily feed intake; ADG, average daily gain; G:F, gain-to-feed ratio; LBP, lipopolysaccharide binding protein.

**Table 3.** Effects of citrulline supplementation on production parameters and circulating lipopolysaccharide-binding protein during heat stress (HS) or pair feeding (PF)

Parameter	Treatment					SEM	*P*-value	Contrasts					
	TNAL	PF-CON	PF-CIT	HS-CON	HS-CIT		Trt	TNAL *vs* PF^1^	TNAL *vs* HS^2^	PF *vs* HS^3^	PF-CIT *vs* PF-CON	HS-CIT *vs* HS-CON	CON *vs* CIT^4^
*Performance*													
FI^5^, kg/2.5 d	5.28^a^	4.03^c^	4.76^ab^	4.08^bc^	4.20^bc^	0.26	<0.01	0.01	<0.01	0.43	0.05	0.72	0.93
ADG, kg	1.02^a^	0.36^b^	0.48^b^	0.82^a^	0.83^a^	0.09	<0.01	<0.01	0.08	<0.01	0.34	0.92	0.34
G:F	0.46^a^	0.24^b^	0.26^b^	0.50^a^	0.49^a^	0.06	0.02	0.01	0.52	<0.01	0.83	0.89	0.60
*Inflammation*													
LBP, µg/mL	12.99	9.06	9.37	11.58	14.37	1.65	0.11	0.08	0.99	0.03	0.89	0.24	0.66

^1^PF-CON and PF-CIT pigs.

^2^HS-CON and HS-CIT pigs.

^3^PF = PF-CON and PF-CIT pigs. HS = HS-CON and HS-CIT pigs.

^4^CON = TNAL, PF-CON, and HS-CON pigs. CIT = PF-CIT and HS-CIT pigs.

^5^Cumulative feed intake during period 2.

^a-c^means with different superscripts significantly differ (*P* ≤ 0.05).

TNAL, thermoneutral ad-libitum; PF-CON, pair-fed control; PF-CIT, pair-fed citrulline; HS-CON, heat stress control; HS-CIT, heat stress citrulline; Trt, treatment; ADG, average daily gain; G:F, gain-to-feed ratio; LBP, lipopolysaccharide binding protein.

Circulating LBP did not differ between CON or CIT-supplemented pigs during P1 (*P* > 0.34; Table 2). During P2, no overall differences were observed for circulating LBP across treatments (*P* > 0.11; Table 3). However, LPB tended to decrease (29%; *P* = 0.08) in PF relative to TNAL pigs and was increased (41%; *P* = 0.03) in HS compared to PF pigs.

The effects of CIT supplementation on intestinal measurements are detailed in Table 4. No differences in jejunum villus height were observed among treatments (*P* > 0.11); however, it decreased in PF relative to TNAL pigs (15%; *P* = 0.03), and this was rescued by CIT supplementation as PF-CIT pigs tended to have increased jejunum villus height compared to PF-CON pigs (16% *P* = 0.10). While jejunum mucosal surface area remained similar among treatments, it decreased in PF (16%; *P* = 0.02) and tended to decrease in HS (11%; *P* = 0.10) compared to TNAL pigs. In the ileum, HS decreased villus height relative to TNAL pigs (10%; *P* = 0.04); however, CIT supplementation improved this metric as HS-CIT tended to have increased ileum villus height compared to HS-CON pigs (367 vs. 327 µm, respectively; *P* = 0.08). Similarly, ileum mucosal surface area decreased (14%; *P* = 0.01) and tended to decrease (8%; *P* = 0.08) in HS relative to TN and PF pigs, respectively. However, CIT supplementation during HS tended to increase mucosal surface area relative to HS-CON pigs (14%; *P* = 0.07). No other effects on villus width, crypt depth, or the VH:CD ratio were observed in the jejunum or ileum across treatments (*P* > 0.29). GC area remained similar among treatments in the jejunum and ileum (*P* > 0.86); however, in the colon, it was decreased in HS-CIT compared to HS-CON pigs (31%; *P* = 0.03). No differences in MPO area were detected in the jejunum, ileum, or colon among treatments (*P* > 0.55; Table 4).

**Table 4.** Effects of citrulline supplementation on production parameters during heat stress (HS) or pair feeding (PF)

Parameter	Treatment					SEM	*P*-value	Contrasts					
	TNAL	PF-CON	PF-CIT	HS-CON	HS-CIT		Trt	TNAL *vs* PF^1^	TNAL *vs* HS^2^	PF *vs* HS	PF-CON *vs* PF-CIT	HS-CON *vs* HS-CIT	CON *vs* CIT^3^
*Jejunum*													
Villus height, µm	454	359	415	419	415	24	0.11	0.03	0.22	0.21	0.10	0.91	0.83
Villus width, µm	144	150	156	148	152	6	0.70	0.24	0.41	0.66	0.45	0.65	0.23
Crypt depth, µm	312	314	309	315	284	20	0.80	0.99	0.62	0.55	0.85	0.29	0.36
VH:CD^4^	1.47	1.18	1.41	1.35	1.55	0.12	0.29	0.25	0.86	0.23	0.18	0.26	0.20
M-index^5^	6.86	5.36	6.12	6.06	6.13	0.37	0.11	0.02	0.10	0.35	0.16	0.89	0.93
GC area, %^6^	14.4	14.4	16.8	14.3	14.6	2.1	0.91	0.65	0.97	0.61	0.43	0.93	0.49
MPO area, %^7^	2.63	3.03	2.66	2.80	3.53	0.70	0.89	0.80	0.53	0.65	0.71	0.47	0.66
*Ileum*													
Villus height, µm	387	374	364	327	367	15	0.11	0.35	0.04	0.17	0.66	0.08	0.85
Villus width, µm	156	164	158	165	166	7	0.83	0.59	0.30	0.53	0.61	0.88	0.91
Crypt depth, µm	233	202	218	197	209	16	0.52	0.25	0.13	0.64	0.48	0.59	0.84
VH:CD^4^	1.79	1.87	1.70	1.71	1.78	0.14	0.91	0.98	0.78	0.78	0.40	0.74	0.69
M-index^5^	5.84^a^	5.51^a^	5.38^a^	4.70^b^	5.35^ab^	0.24	0.04	0.20	0.01	0.09	0.70	0.07	0.95
GC area, %^6^	33.5	27.9	29.9	27.6	26.0	5.11	0.86	0.47	0.29	0.69	0.78	0.84	0.72
MPO area, %^7^	1.75	2.58	1.55	2.16	2.32	0.60	0.74	0.67	0.51	0.77	0.23	0.86	0.67
*Colon*													
GC area, %^6^	56.4	56.3	51.6	64.8	44.4	6.2	0.24	0.75	0.81	0.92	0.60	0.03	0.06
MPO area, %^7^	0.71	1.17	0.56	0.93	1.28	0.34	0.55	0.72	0.36	0.49	0.22	0.48	0.95

^1^PF-CON and PF-CIT pigs.

^2^HS-CON and HS-CIT pigs.

^3^CON = TNAL, PF-CON, and HS-CON. CIT = PF-CIT and HS-CIT.

^4^Villus height-to-crypt depth ratio.

^5^Mucosal surface area expressed as an M-index as described by Kisielinski et al. (2002).

^6^Goblet cell (GC) area; expressed as a percentage of epithelial area.

^7^Percentage of positive myeloperoxidase (MPO) relative to total stained area.

^a,b^Means with different superscripts significantly differ (*P* ≤ 0.05).

TNAL, thermoneutral ad-libitum; PF-CON, pair-fed control; PF-CIT, pair-fed citrulline; HS-CON, heat stress control; HS-CIT, heat stress citrulline; Trt, treatment.

## Discussion

HS negatively impacts almost every key performance indicator and is thus a significant financial burden for the U.S. swine industry (St. Pierre et al., 2003; Ross et al., 2015). HS affects productivity indirectly by reducing FI and directly by altering multiple systems, including physiology, metabolism, and the immune system (Baumgard and Rhoads, 2013; Mayorga et al., 2020). HS causes peripheral vasodilation at the expense of blood flow to splanchnic tissues. Reduced nutrient and oxygen supply leads to GIT hypoxia, which can negatively affect morphology, impair nutrient absorption, and cause hyperpermeability (Hall et al., 2001; Pearce et al., 2013a, b, 2015). Intestinal barrier dysfunction can lead to antigen (i.e., endotoxin) infiltration and immune activation, which comes at a large energetic cost (Johnson, 2012; Kvidera et al., 2017b; Huntley et al., 2018). Furthermore, concurrent immune activation and vasodilation put a strain on NO synthase (**NOS**) and Arg availability. Endothelial NOS is essential for maintaining vascular homeostasis (e.g., vasodilation and inhibition of platelet aggregation). Alternatively, inducible NOS is stimulated by inflammation and is involved in the immune response via high NO production, mainly by macrophages (Wijnands et al., 2015). Arg is the amino acid precursor for NO synthesis. Consequently, increased inducible NOS activity may hamper endothelial NOS function by limiting Arg supply, especially in situations when both NOS are needed (Asgeirsson et al., 2011). Contributors to the Arg pool include dietary intake, skeletal muscle proteolysis, and de novo synthesis from CIT. Citrulline is produced by small intestine enterocytes and is the precursor for ~60% of the body’s de novo Arg (Yu et al., 1996). Circulating levels of both CIT and Arg decrease during HS (Chowdhury et al., 2014; Morales et al., 2016). This is likely a combination of decreased amino acid consumption, increased Arg metabolism, and decreased alimentary tract mass and dysfunction, leading to decreased CIT and Arg absorption and de novo synthesis (i.e., decreased enterocyte numbers and function; Morales et al., 2016). In fact, CIT is often used as a marker of functional GIT mass because it is produced by healthy enterocytes and is a non-protein amino acid (Wells et al., 2017). Interestingly, supplemental CIT can improve Arg status more than Arg itself (Wijnands et al., 2012; Agarwal et al., 2017), mainly because CIT largely bypasses splanchnic extraction and is efficiently converted to Arg in the kidney (Cynober, 1994; Bahri et al., 2013). Furthermore, dietary Arg can cause GIT distress and diarrhea (Grimble, 2007), making CIT a more attractive feed additive option to increase the Arg pool. Thus, study objectives were to evaluate if supplemental CIT could benefit thermal and gut morphology parameters during HS and feed restriction in growing pigs.

HS was successfully induced in the current study, as indicated by increased Tr, Ts, and RR. Interestingly, supplementing CIT during HS slightly decreased Tr and RR; however, this difference was only evident in the first 30 h of HS and diminished thereafter. This observation agrees with a previous study where CIT supplementation decreased RR in lactating sows during summer (Liu et al., 2019). Additionally, supplemental CIT (either dietary or orally administered) ameliorated the increase in rectal temperature in heat-stressed chickens (Chowdhury et al., 2017, 2021a, b; Uyanga et al., 2021). The exact mechanisms by which CIT influences body temperature are not yet entirely clear. Increased skin vasodilation and heat dissipation, presumably via NO, are likely mediators of this response (Mathai et al., 1997; Kellogg Jr et al., 1998). However, independent of NO, CIT has been shown to reduce body temperature and influence thermotolerance in chicks, suggesting other factors might contribute to the thermoregulatory function of CIT (Chowdhury et al., 2017; Chowdhury, 2023). For instance, Chowdhury et al. (2021b) observed that orally administered CIT in heat-stressed chickens decreased FI and reduced circulating T_3_ (i.e., triiodothyronine), a key marker of energy metabolism. Considering that nutrient digestion and absorption generate metabolic heat, decreased FI represents an efficient strategy to decrease body temperature during HS (Collin et al., 2001). Nevertheless, this evidence, coupled with our observations, suggests that CIT supplementation effectively reduces some of the physiological strain of HS, but further research is needed to better understand the mechanisms (and repeatability) behind this response.

Another potential mode of action by which CIT may help alleviate HS is by improving gut microcirculation, leading to enhanced gut health and subsequently reduced inflammation. Citrulline supplementation has been shown to improve intestinal barrier function during hypoxia and mucositis (Chapman et al., 2012; Antunes et al., 2016) and can improve intestinal microcirculation during endotoxemia and exercise (Wijnands et al., 2012; van Wijck et al., 2014). As mentioned previously, poor microcirculation and subsequent hypoxia could potentially contribute to the etiological cause of intestinal barrier dysfunction during HS (Kregel et al., 1988; Hall et al., 2001). Various studies have shown positive effects of Arg on intestinal barrier function during HS (Varasteh et al., 2018; Xia et al., 2019; Yi et al., 2020), indicating this is likely a NO-mediated effect. In the current study, we observed distinct histological and morphological alterations, such as shorter villi and decreased mucosal surface area, that were more evident in the jejunum and ileum when compared to the colon. Each stressor had different impacts on intestinal morphology; nutrient restriction primarily affected the jejunum, while the effects of HS were more pronounced in the ileum. It remains uncertain whether the proximal sections of the small intestine are more susceptible to injury from either nutrient restriction or HS when compared to the colon. Evidence from a poultry study suggests regional differences within the small intestine in response to HS, with more apparent alterations occurring in the ileum compared to the jejunum (Varasteh et al., 2015). This contradicts our previous observations in heat-stressed pigs, where the ileum seems to “heal” more quickly from HS than the jejunum (Abuajamieh et al., 2018). Unlike the colon, the small intestine has a high metabolic rate, making it more susceptible to hypoxia during a HS event (Sherratt, 1968; Novosad et al., 2013). Furthermore, feed restriction itself is a stressor that can directly affect intestinal integrity in pigs (Pearce et al., 2013a) and other species (Rodriguez et al., 1996; Welsh et al., 1998; Boza et al., 1999; Horst et al., 2020). Fasting reduces mucosal surface area, villus height, cell numbers, cell proliferation, and cell migration rates, coupled with increased rates of cell loss, apoptosis, and mucin depletion, altogether contributing to increased intestinal permeability (Sherman et al., 1985; Ferraris and Carey, 2000; Chappell et al., 2003). While it is unlikely that feed restriction reduces blood flow enough to cause hypoxia-induced damage, withholding feed likely creates psychological stress associated with inflammation and intestinal hyperpermeability (Overman et al., 2012; Pohl et al., 2017; Proudfoot et al., 2018). This is thought to happen via the release of corticotropin-releasing factor and its stimulatory effect on intestinal mast cell activation, which negatively impacts barrier function (Albert-Bayo et al., 2019). Different physiological effects of each stressor (hypoxia vs. mast cell activation) may help explain why different intestinal sections were affected herein. Considering these regional variations along the GIT, it is imperative to better characterize the effects of nutrient restriction and HS on gut health, as changes in intestinal architecture, permeability, and the ensuing inflammatory response to these stressors might pathologically differ between the proximal and the distal sections of the gut.

Interestingly, there seem to be differences in how CIT affects the gut depending on the stressor. Feeding CIT during HS had no positive effects on the jejunum. However, supplementing CIT during HS improved villus height and mucosal surface area in the ileum by 12 and 14%, respectively. Contrary to our hypothesis and previous results in a rat ischemia-reperfusion model (Gou et al., 2011), the GC area in the colon was depleted 31% by supplementing CIT during HS compared to HS-CON. Reasons for this are unclear, but could be detrimental as GC area in HS-CIT animals was reduced to even 21% less than TNAL animals during a time when intestinal integrity is compromised and mucin is likely an important barrier. However, HS-CON animals did have a numerical increase (~15%) in GC area relative to TNAL (*P* = 0.34) and therefore this reduction by CIT could potentially represent a return of colon tissue to more normal mucin-producing levels. Conversely, supplementing CIT during pair-feeding (i.e., feed restriction) had only moderate improvements in jejunum villus height. Previous studies have shown improved histopathological scores in the jejunum, but not the ileum, of mice supplemented with CIT during a mucositis challenge (Antunes et al., 2016). During HS, Arg improved jejunum villus height, VH:CD, and tight junction expression in rats (Xia et al., 2019). In a jejunal cell culture model, both Arg and CIT improved barrier function and preserved tight junction proteins during hypoxia, and inhibiting NO synthase negated this effect (Chapman et al., 2012). It is unclear why there are such regional discrepancies in the apparent positive effect of Arg and/or CIT supplementation across studies, but it may depend on the HS protocol, species, or the stress model employed. Furthermore, as previously mentioned, regional differences in anatomy and physiology along the GIT, including epithelial structure, nutrient absorption, microbial load, and immune function, might also influence the response to CIT supplementation during HS or nutrient restriction. Regardless of the mechanism, understanding how CIT supplementation positively influences intestinal architecture along the GIT is critical in developing nutritional strategies to minimize the impacts of HS and feed restriction on animal health and productivity.

Contrary to our expectations, no differences in intestinal MPO were observed for either HS or feed restriction. This study is limited in that intestinal MPO was the only direct measure of intestinal inflammation, and the study would have benefitted from additional measures of intestinal inflammation. The lack of difference in MPO could be attributed to the timing of HS onset. McConnico et al. (1999) observed that in horses, MPO activity increases during the first 16 h after injury to the mucosal barrier and declines thereafter. It is possible that the initial influx of neutrophils to the intestine during HS could be relatively short-lived as indicated by previous studies observing increased MPO activity at 24 h of HS in pigs (Pearce et al., 2013b) but not after 7 d of HS in calves (Yu et al., 2023). A HS duration of 2.5 d was selected to mimic practical conditions of a heat wave (i.e., 1 to 2 d of high environmental temperatures) sufficient enough to induce a stress response yet not allow for long-term physiological adaptations to develop. The HS protocol was cyclical, and 2.5 d was selected so that animals were sacrificed during peak heat (~12 h after heat was increased in the AM) when blood flow was likely to be most distributed to the periphery. The study does not represent chronic effects of HS on intestinal parameters, and results of this trial warrant further research to better understand the differences in chronic vs. acute HS on intestinal histology and inflammation.

The liver produces acute phase proteins such as LBP as an inflammatory mediator in response to pathogen-associated molecular patterns, and these are often used as a proxy for systemic inflammation (Zweigner et al., 2006). Presumably, the endotoxin source of inflammation in the case of this HS study would likely be intestinal barrier hyperpermeability. Contrary to stimulating the immune system through an endotoxin challenge, HS uniquely seems to suppress inflammatory markers such as LBP, which then increase during recovery (Pearce et al., 2013a, 2015; Abuajamieh et al., 2018). It is unclear why this pattern of LBP occurs, but it may be due to suppression of LBP synthesis by heat shock proteins or glucocorticoids (Chen et al., 2006). Although pair-feeding appeared to decrease circulating LBP in the current study, it was maintained at the level of TNAL pigs during HS. Reasons for this distinctive response in LBP between nutrient-restricted and heat-stressed pigs are not clear. However, LBP enhances the innate immune response to bacterial components; thus, elevated concentrations during HS may contribute to initiating an effective immune response. Citrulline supplementation did not alter circulating LBP levels during PF or HS; however, as it was not the focus of the current study, only one inflammatory marker (at one-time point) during HS and PF was measured. It would be worthwhile to investigate the effects of CIT on inflammation in more focused studies with multiple inflammatory markers over time.

The effects of feed restriction and HS on intestinal permeability demonstrate the fragility of intestinal barrier function, and dietary strategies to improve intestinal health and function emerge as promising strategies to alleviate the negative effects of these stressors. However, the current study has limitations, as we relied on intestinal morphology as an indirect measure of intestinal barrier health and did not directly measure intestinal hyperpermeability. Furthermore, both basal diet and plasma levels of CIT and Arg were not measured. Understanding the Arg content of a typical commercial diet and how it, coupled with CIT supplementation, might influence intestinal health is crucial. It is plausible that decreased Arg availability during HS is a protective mechanism to prevent excessive NO production and limit inflammation during immune activation, and supplementing too much CIT or Arg could lead to detrimental effects. Further investigations into the optimal supplemental CIT dose, its mode of action, and the temporal pattern of inflammation are warranted. Alternatively, conducting larger-scale production studies involving varying HS durations may help determine if physiological effects can translate into significant performance differences and if there is a minimum or maximum HS duration during which CIT supplementation proves advantageous.

## Conclusion

Supplementing CIT reduced RR and rectal temperature during HS. Interestingly, feed restriction and HS differentially affected jejunum and ileum morphology by decreasing villus height and reducing mucosal surface area. Supplementing CIT rescued some of these metrics, but the effects were dependent on intestinal region and stressor type. While our observations suggest a potential benefit of CIT supplementation in mitigating the physiological strain induced by the thermal stress and improving gut health during HS and feed restriction, further investigation is warranted to validate these effects.

## Abbreviations

ADG: average daily gain
AL: ad-libitum
BW: body weight
c.: crypt
CIT: citrulline
CON: control
FI: feed intake
G:F: gain to feed ratio
GC: goblet cell
GIT: gastrointestinal tract
H&E: hematoxylin and eosin
HS: heat stress
HS-CON: heat stress control
HS-CIT: heat stress citrulline
LBP: lipopolysaccharide binding protein
MPO: myeloperoxidase
NO: nitric oxide
OS: nitric oxide synthase
P1: period 1
P2: period 2
PAS: periodic acid-Schiff
PF: pair-fed
PF-CON: pair-fed control
PF-CIT: pair-fed citrulline
RR: respiration rate
TN: thermoneutral
TNAL: thermoneutral ad-libitum control
Tr: rectal temperature
Ts: skin temperature
v.: villus
VH:CD: villus-height-to-crypt-depth-ratio
